# The Influence of Various Distraction Stimuli on Affective Responses during Recumbent Cycle Ergometry

**DOI:** 10.3390/sports4020021

**Published:** 2016-03-23

**Authors:** Paul C. Miller, Eric E. Hall, Elizabeth K. Bailey

**Affiliations:** 1 Department of Exercise Science, Elon University, Elon, NC 27244, USA; ehall@elon.edu; 2 Department of Health and Human Performance, Elon University, Elon, NC 27244, USA; ebailey@elon.edu

**Keywords:** exercise, distraction, feeling scale, affect

## Abstract

(1) Background: Acute bouts of exercise have been associated with affective changes. Exercise supplemented with distraction may divert attention from unpleasant feelings commonly associated with exercise to more pleasant feelings. The purpose of this study was to compare affective responses to exercise with and without distraction. (2) Methods: 25 individuals volunteered for this investigation and completed all three conditions. This study included three 30 min cycle ergometry exercise conditions, a control condition with no stimuli and two test conditions; one supplemented with a self-selected video and the other self-selected music. The Feeling Scale (FS) was administered prior to, every 10 min during, immediately following, and 10 min post exercise. (3) Results: These data demonstrate a significant condition effect for FS during exercise. The condition effect was due to FS being greater in the video and distraction conditions. There was no time by condition interaction seen during exercise. (4) Conclusion: These data indicate that distraction may be effective in supporting a more pleasant exercise experience and could potentially increase exercise adherence.

## 1. Introduction

Physical inactivity and adherence to exercise continue to be on-going problems in contemporary society. Recent evidence suggests that 44% of US adults are aerobically active and 28% are highly active [1] according to the *2008 Physical Activity Guidelines for Americans* [2]. These guidelines call for 150 min of moderate intensity physical activity per week.

A recent meta-analysis found that traditional aspects of physical activity such as frequency, intensity, duration and mode play almost no role in exercise adherence [3]. The authors advocate that other variables such as personality, social cognitive, socioeconomic and environmental factors need to be taken into consideration. This focus on behavioral characteristics related to physical activity participation is warranted.

One such nontraditional factor to consider is affective responses to exercise [4,5]. In the most recent American College of Sports Medicine position stand concerning physical activity guidelines it is recommended that measures of pleasantness such as the Feeling Scale (FS) can be used to regulate and monitor exercise intensity [6]. Recent studies have found affective responses to exercise to be related to future exercise behavior [7,8,9,10]. One such study by Williams *et al.* found that a 1-unit increase on the Feeling Scale [11] during moderate intensity exercise was related to an additional 38 min of moderate intensity at 6 months follow-up and 41 additional min at 12 months [9].

Factors influencing exercise affect, such as the exercise environment, should be considered in the development of exercise programs. There are a variety of ways to manipulate exercise environment. One such way to influence the environment would be to manipulate what an individual listens to or watches during exercise. Auditory and visual distraction during exercise may be a viable strategy to alter one’s perception of unpleasant feelings often associated with intense exercise [12,13,14,15,16,17,18,19,20,21]. Therefore, performing exercise with distraction stimuli may divert an individual’s attention from feelings of fatigue and discomfort to more pleasant feelings [22].

Studies suggest that distraction techniques, such as music, increase work output during exercise [20,23] and improve post-exercise feeling states [12,14,19,21]. However, the most effective distraction modality to mediate the negative feelings often associated with exercise remains elusive. Recent research has shown that an enjoyable video augments the positive response following exercise [21], but other research has shown that music only [19] or music and video [12,19] show more positive affective responses. Many of these previous studies select the exercise intensity and distraction materials (music and/or video) for the participants [12,19,20,21]. However, Williams theorizes that self-paced exercise will lead to greater affective benefits and exercise adherence [24]. Moreover, recent reviews by Karageorghis and Priest suggest that the effects of listening to music during exercise are most beneficial when the exercise is self-paced [25,26], but less is known about the use of videos. Many previous studies use music videos for their stimuli [12,19,20] but few use television shows or other forms of visual stimuli [21]. Therefore, the purpose of this study was to compare individuals’ affective response to exercise at a self-selected intensity when using self-selected visual distraction (video), self-selected auditory distraction (music), and no distraction. This study strived to achieve a high level of ecological validity by utilizing self-selected distraction stimuli and by allowing the participants to choose the exercise intensity.

## 2. Results

A 3 (condition: music, video, no distraction) by 3 (time: 10 min, 20 min, 30 min) repeated measures general linear model (RM-GLM) for ratings of perceived exertion (RPE) showed a significant main effect for time [F (2, 23) = 31.56, *p* < 0.001], but no condition effect [F (2, 23) = 1.44, *p* = 0.26] or condition × time interaction [F (4, 21) = 0.80, *p* = 0.54] (See Table 1).

A 3 (condition: music, video, no distraction) by 3 (time: 10 min, 20 min, 30 min) repeated measures general linear model (RM-GLM) for heart rate (HR) also showed a main effect for time [F (2, 23) = 10.45, *p* = 0.001], but no condition effect [F (2, 23) = 0.31, *p* = 0.74] or condition × time interaction [F (4, 21) = 0.57, *p* = 0.69] (See Table 2).

A 3 (condition: music, video, no distraction) by 3 (time: 10 min, 20 min, 30 min) RM-GLM for FS showed a significant main effect for condition [F (2, 23) = 6.16, *p* = 0.007]. However, there was not a significant time [F (2, 23) = 2.04, *p* = 0.153] or condition x time interaction [F (4, 21) = 0.73, *p* = 0.584]. The pairwise comparisons revealed that the condition effect was due to FS being greater in the music (*p* = 0.006) and video (*p* = 0.004) distraction conditions (See Table 3).

A 3 (condition: music, video, no distraction) by 3 (time: pre-ex, post ex-0 min, post ex-10 min) RM-GLM for FS showed a significant main effect for time [F (2, 23) = 7.83, *p* = 0.003] with FS post-10 being significantly higher than FS pre (*p* = 0.039) and FS post-0 (*p* = 0.008). However, there was not a significant condition [F (2, 23) = 1.93, *p* = 0.168] or condition x time interaction [F (4, 21) = 2.19, *p* = 0.105] (See Table 4).

## 3. Discussion

In the current study, no differences were seen in self-selected exercise intensity as indicated by RPE and HR in the three conditions. Finding no differences in perceived intensity and work is contrary to what would be expected according to Karageorghis and Priest [25,26]. The fact that the present study did not find a difference in perceived intensity and work may be due to the fact that the participants may have had pre-conceived goals for the exercise session which influenced the self-selected intensity more so than the effect of the condition. In other words, the participants may have selected an exercise pace that they believed matched their ability to complete the exercise trial. However, it was found that the participants exercised in the light to moderate range as classified by RPE (ranged 10.6–13.1) and HR (ranged in approximately 57%–65% of percent HR_max_). This suggests that the participants were exercising at an intensity that would be consistent with recommendations aimed at deriving health benefits and consistent with guidelines for physical activity [6].

The affective responses to exercise in the present study are similar to previous research that has shown improvements in affect following exercise, post 10 min, no matter the intensity or condition [4]. Additionally, previous research suggests that the use of distraction may allow for a more pleasant exercise experience [12,13,14,16,19,21,27]. The findings of the present study are fairly consistent with these as well. There were significant differences by condition in affective responses as measured by the FS. In the two distraction conditions, video and music, participants reported feeling more positive than when no distraction was given. This may be an indication that for recreational exercisers, the use of either self-selected music or video distraction during exercise resulted in a similarly more pleasant exercise experience while still allowing for the exercise to be done at an intensity that would support fitness and health benefits. Additionally, it should be noted that the no distraction group reported declines in affect during the exercise while exercise affect was maintained during the music and video conditions. This may be due to where the exerciser focused their attention, on the exercise itself or on music or a video.

Emmons and Diener found that people are more likely to repeat activities that make them feel good during the activity [28]. In regards to exercise behavior, studies have found that affective responses to exercise are linked to future exercise behavior [7,8,9,24]. As mentioned previously, Williams *et al.* found that a 1-point difference on FS during moderate intensity exercise had influences on behavior at 6 and 12 months [9]. This 1-point difference is nearly the amount of difference observed between the distraction and no distraction conditions in this study. Therefore, using distraction during exercise might be an effective behavior modification technique for facilitating ongoing exercise participation. The uniqueness of this study is that the participants were able to self-select the music and video they watched during exercise as well as their exercise intensity allowing for increased ecological validity for the present study.

The strength of this study was the focus on ecological validity related to the choice of distraction stimuli and the self-selected exercise intensity. While these strengths exist, there are some limitations including the lack of standardization regarding the content of the distraction selected and the participants’ exercise history.

## 4. Materials and Methods

### 4.1. Participants

Twenty-five healthy, college students (14 male, *M* age = 20.3 ± 1.4 years, *M* BMI = 24.3 ± 2.7; 11 female, *M* age 20.3 ± 1.4 years, *M* BMI = 21.9 ± 1.4) volunteered to participate in this study. Prior to participation, individuals completed an informed consent and a Physical Activity Readiness Questionnaire [29]. No prior exercise experience was required to participate. All procedures used in this study were approved by the University’s Institutional Review Board.

### 4.2. Measures

Affect was assessed by the Feeling Scale (FS) [11]. The FS is an 11-point, single-item, bipolar rating scale. The FS is routinely used to assess affect during exercise because it has been found to be valid and reliable [30]. The FS scale ranges from –5 (“Very Bad”) to +5 (“Very Good”). Exercise intensity was assessed by recording heart rate (HR) and ratings of perceived exertion (RPE) [31]. Heart rate was measured using a Polar™ Heart Rate Monitor and RPE was assessed using Borg’s 20-point scale which ranges from 6 “no exertion at all” to 20 “maximal exertion”.

### 4.3. Procedures

This study utilized a repeated measures design. Each participant completed all three 30 min recumbent cycle ergometry exercise sessions. The exercise sessions were separated by one week and were performed at the same time of day. Each exercise session utilized a different distraction condition; a control condition with no stimuli, a video stimulus, and a music stimulus. Both video and music stimuli were self-selected by the participants and all conditions were administered in a random order. Participants were asked to bring a video of a movie or a television series and music selection of their choice to each session so they wouldn’t know what condition they would be assigned.

The participant was seated on the cycle ergometer and the seat was adjusted to fit the height of the individual. The participant was then instructed to pedal the cycle ergometer at an intensity as if they were exercising for fitness. The first 5 min of exercise served as an acclimation/warm-up segment. They were permitted to freely change intensity within the first 5 min of exercise and then only every 5 min thereafter. Participants were allowed to adjust their exercise intensity every 5 min to allow them to reach a steady state at that intensity before making any changes. The exercise bout was 30 min long followed by a 10 min passive recovery, sitting on the cycle ergometer. The cycle ergometer was placed in front of a television that was used to play the music or video that was brought to the laboratory by the participant. Participants were allowed to have the volume adjusted to the desired level. The participants were not given any additional instructions about viewing or listening to the television once the exercise was started. In the control condition, the participant looked at an unpowered television. The individual wore a Polar Heart Rate Monitor for the duration of each exercise session and throughout the 10 min passive recovery. Affect was measured prior to the onset of exercise, every 10 min during exercise, immediately following, and 10 min post exercise. Exercise intensity was assessed by recording RPE and HR every 10 min throughout the exercise bout. This protocol was completed three times, once for each treatment condition.

### 4.4. Statistical Analysis

A Condition (3: music, video, no distraction) by Time (10 min, 20 min, 30 min) Repeated Measures General Linear Model (RM-GLM) was used to determine main and interaction effects of condition and time (within-subjects repeated measure) for each of the variables (*i.e*., RPE, HR and FS). Another Condition by Time (pre, post-0, post 10 min) Repeated Measures General Linear Model (RM GLM) was used to determine main and interaction effects of condition and time (within-subjects repeated measure) for the Feeling Scale. The analyses for FS were run separately because during exercise responses are thought to be different from pre and post-exercise responses [4]. Fisher’s Least Significant Difference (LSD) test was performed when main effects were found.

## 5. Conclusions

The use of distraction stimuli may assist an individual in overcoming feelings of discomfort and unpleasantness commonly elicited by exercise and frequently offered as reasons for discontinuation of exercise. The results of this investigation suggest that the addition of music or video distraction during exercise may allow an individual to focus their attention on the distraction rather than on unpleasant or uncomfortable feelings related to their exercise. This may lead to a more pleasant exercise experience and potentially impact future exercise adherence [7,8,9,10]. Future research may want to examine the efficacy of using distraction as an exercise intervention technique and the influence this may have on adherence to physical activity.

## Figures and Tables

**Table 1:** During exercise ratings of perceived exertion (RPE) responses over time and by condition.

Time	Music	Video	No Distraction
RPE 10 min	10.8 ± 1.8	10.4 ± 1.9	10.6 ± 2.1
RPE 20 min	12.4 ± 2.0	11.7 ± 2.1	12.4 ± 2.1
RPE 30 min	13.1 ± 2.4	12.6 ± 2.6	13.0 ± 2.6

**Table 2:** During exercise heart rate (HR) responses over time and by condition.

Time	Music	Video	No Distraction
HR 10 min	113.9 ± 28.2	113.6 ± 22.4	114. 7 ± 18.9
HR 20 min	123.2 ± 23.0	119.3 ± 26.2	120.8 ± 18.3
HR 30 min	130.1 ± 27.8	128.1 ± 27.4	126.5 ± 20.4

**Table 3:** During exercise Feeling Scale (FS) responses over time and by condition.

Time	Music *	Video **	No Distraction
FS 10 min	2.7 ± 1.2	2.5 ± 1.6	2.2 ± 1.4
FS 20 min	2.4 ± 1.5	2.4 ± 1.5	1.7 ± 1.5
FS 30 min	2.4 ± 1.6	2.5 ± 1.8	1.8 ± 1.7

Notes: * Significant condition effect compared to no distraction (*p* = 0.006); ** Significant condition effect compared to no distraction (*p* = 0.004).

**Table 4:** Pre and post-exercise Feeling Scale (FS) responses over time and by condition.

Time	Music	Video	No Distraction
FS pre-ex	2.7 ± 1.5	2.3 ± 2.1	2.3 ± 1.8
FS post ex-0 min	2.5 ± 1.6	2.4 ± 1.8	1.8 ± 1.8
FS post ex-10 min	3.3 ± 1.1	2.7 ± 1.5	2.8 ± 1.4

## Abbreviations

The following abbreviations are used in this manuscript:

FS	Feeling Scale
BMI	body mass index
RPE	ratings of perceived exertion
RM-GLM	repeated measures general linear model
HR	heart rate
